# Evidence quality assessment of acupuncture intervention for stroke hemiplegia: an overview of systematic reviews and meta-analyses

**DOI:** 10.3389/fneur.2024.1375880

**Published:** 2024-04-30

**Authors:** Maoxia Fan, Bo Zhang, Chen Chen, Runmin Li, Wulin Gao

**Affiliations:** ^1^First Clinical Medical College, Shandong University of Traditional Chinese Medicine, Jinan, China; ^2^Dongying People’s Hospital (Dongying Hospital of Shandong Provincial Hospital Group), Dongying, China; ^3^Department of Geriatric Medicine, Affiliated Hospital of Shandong University of Traditional Chinese Medicine, Jinan, China

**Keywords:** acupuncture, stroke hemiplegia, systematic reviews, meta-analyses, overview

## Abstract

**Objective:**

Summarize the conclusions of the systematic review/meta-analysis of the clinical efficacy of acupuncture for stroke hemiplegia, and evaluate its methodological quality and the quality of evidence.

**Methods:**

Two researchers searched and extracted 8 databases for systematic reviews (SRs)/meta-analyses (MAs), and independently assessed the methodological quality, risk of bias, reporting quality, and quality of evidence of SRs/MAs included in randomized controlled trials (RCTs). Tools used included the Assessment of Multiple Systematic Reviews 2 (AMSTAR-2), the Risk of Bias in Systematic (ROBIS) scale, the list of Preferred Reporting Items for Systematic Reviews and Meta-Analysis (PRISMA), and the Grading of Recommendations Assessment, Development, and Evaluation (GRADE) system. The search time is from database building to July 2023.

**Results:**

A total of 11 SRs/MAs were included, including 2 English literature and 9 Chinese literature, with all study sites in China. AMSTAR-2 evaluation results showed that the methodological quality of 11 articles was rated as very low quality; Based on the ROBIS evaluation results, the SRs/MAs was assessed as a high risk of bias; According to the results of the PRISMA checklist evaluation, most of the SRs/MAs reports are relatively complete; according to GRADE system, 42 outcomes were extracted from the included SRs/MAs for evaluation, of which 1 was rated as high-quality evidence, 14 as moderate-quality evidence, 14 as low-quality evidence, and 13 as very low-quality evidence.

**Conclusion:**

The available evidence indicates that acupuncture has certain clinical efficacy in the treatment of stroke hemiplegia. However, there are still some limitations to this study, such as the lower quality of SRs/MAs methodologies and evidence included, and more high-quality studies are needed to verify them.

## 1 Introduction

Stroke is a common cerebrovascular disease, also often known as “stroke” (1), belongs to a common clinical disease. It is characterized by high morbidity, high mortality rate, high disability rate and many complications (2, 3), and the prevalence rate is increasing year by year (4). With the improvement of medical technology level, the mortality of stroke patients decreased significantly, but still about 80% of patients in the disease recovery will leave a variety of different degrees of neurological and limb dysfunction (5), including hemiplegia is one of the common sequelae of stroke (6), not only affect the quality of life, also bring heavy burden to the family and society. Therefore, taking timely and effective rehabilitation treatment to promote the recovery of limb movement function of stroke patients and improve the self-care ability has become an important problem to be solved in clinical practice.

Acupuncture therapy for stroke and its sequelae has received much attention due to its long history and unique treatment experience (7). Related studies have found that acupuncture can improve the clinical performance of patients with by promoting cerebral blood circulation, affecting astrocytes, blood rheology, and neuronal plasticity (8, 9). In recent years, a large number of clinical randomized controlled trials showed that acupuncture is effective in the treatment of stroke hemiplegia (10, 11). At present, several systematic reviews (SRs)/meta-analyses (MAs) have comprehensively analyzed the relevant clinical data, which provides a theoretical basis for the research of acupuncture for stroke hemiplegia. However, the quality of the evidence obtained by the secondary institute is influenced by the quality of the original data and the subjective factors of the researchers, and whether the method evaluation is standardized, objective and fair, and can fully and effectively evaluate the clinical efficacy of acupuncture in stroke hemiplegia. To provide higher evidence support, this study will re-evaluate the existing SRs/MAs of acupuncture for the treatment of stroke hemiplegia at home and abroad. Using tools including the Assessment of Multiple Systematic Reviews 2 (AMSTAR-2) (12, 13), the Risk of Bias in Systematic (ROBIS) (14) scale, the list of Preferred Reporting Items for Systematic Reviews and Meta-Analysis (PRISMA) (15, 16), and the Grading of Recommendations Assessment, Development, and Evaluation (GRADE) (17–19) system for methodology, risk of bias, reporting quality and evidence quality rating. The existing research results are systematically summarized in order to provide a more accurate evidence-based basis for subsequent clinical decisions (20).

## 2 Materials and methods

The methodology of this overview follows the Cochrane Handbook, and the report of this overview is in line with the Preferred Reporting Items for Systematic Reviews and Meta-Analyses (PRISMA) 2020 checklist. This overview has been registered with the PROSPERO website (Registration number: PROSPERO CRD42024495984).

### 2.1 Inclusion and exclusion criteria

Contributions to acupuncture treatment of post-stroke paralysis based on existing RCTs. The criteria for inclusion of SRs/MAs in this overview are as follows: (1) Based on the SRs/MAs of randomized controlled trials (RCTs), the published language was in Chinese or English. (2) Patients were diagnosed with paralysis after stroke, regardless of the type of stroke (hemorrhagic stroke or ischemic stroke), the diseased brain area, the affected limb, gender, age, ethnicity, and nationality. (3) The intervention measures in the treatment group were acupuncture or acupuncture combined with other treatments, while the control group received non-acupuncture therapy. (4) The main outcome measures are: ① fugl-meyer as-sessment of low limb (FMA-L); ② Barthel Index (BI); ③ Clinic Neurological Function Deficit Scale (NDS); ④ The Modified Ashworth Spasticity Rating Scale score (MAS); ⑤ Clinical effectiveness; ⑥ Brunnstorm stage score; ⑦ The Clinical Spasticity Index score (CSI); ⑧ The Berg Balance Scale score; ⑨ functional comprehensive assessment score (FCA).

Exclusion criteria: Repeated published literature, animal studies, dissertations, conference articles, systematic review protocols and literature where raw data cannot be extracted.

### 2.2 Search strategy

A computer search was conducted across China National Knowledge Network (CNKI), Wanfang database, VIP database, Chinese biomedical literature database (CBM) and PubMed, EMbase, Cochrane Library and other English databases, the search time from the establishment of the database to December 31, 2023. In addition, we manually supplemented the references and grey literature of the included studies. The retrieval method adopts the combination of subject words and free words. Key words include acupuncture, electric acupuncture, needling, head needle, body needle, flying needle, fire needle, acupuncture with warmed needle, acusector, prod, Post-stroke hemiplegia, stroke hemiplegia, cerebral infarction hemiplegia, spastic hemiplegia, flaccid hemiplegia, systematic review, meta analysis. Taking CNKI as an example, the specific search strategy is as follows: SU = (“acupuncture” + “electric acupuncture” + “needling” + “head needle” + “acupuncture with warmed needle” + “acusector” + “prod”) and SU = (“Post-stroke hemiplegia” + “stroke hemiplegia” + “cerebral infarction hemiplegia” + “spastic hemiplegia” + “flaccid hemiplegia”) and SU = (“systematic review” + “meta analysis”); Search strategy for the PubMed database see Table 1.

**Table 1:** Search strategy for the PubMed database.

Query	Search terms
#1	“Acupuncture” OR “electric acupuncture” OR “head needle” OR “body needle” OR “flying needle” OR “fire needle” OR “needling” OR “acupuncture with warmed needle” OR “acusector” OR “prod”
#2	“stroke” [mesh]
#3	“Post-stroke hemiplegia” OR “stroke hemiplegia” OR “cerebral infarction hemiplegia” OR “spastic hemiplegia” OR “flaccid hemiplegia”
#4	#2 OR #3
#5	Meta-Analysis as Topic
#6	“Systematic review” OR “meta-analysis” OR “meta analysis” OR “meta-analyses” OR “review, systematic” OR “systematic reviews”
#7	#5 OR #6
#8	#1 AND #4 AND #7
#9	#5 AND #8

### 2.3 Literature screening and data extraction

Two researchers (MX-F and RM-L) independently screened and extracted the literatures, and cross-checked them. Any disagreement during this process were subject to discussion and negotiation or the decision of a third expert (WL-G). The extracted literature information included: Authors, publication year, nationality, sample size, intervention measures, quality assessment tools and main conclusions.

### 2.4 Quality assessment

Two researchers (MX-F and RM-L) independently assessed the methodological and evidence quality of the included SRs/MAs, Any discrepancies were resolved by consensus or adjudication by a third author (WL-G).

#### 2.4.1 Methodological quality assessment

The methodological quality of the included SRs/MAs was evaluated using the Assessment System for Evaluating Methodological Quality 2 (AMSTAR-2). The AMSTAR-2 scale contains 16 items that can be answered with a “yes,” “partially yes” or “no.” Item 1: Whether the research question and inclusion criteria include the elements of PICO; 2: Whether the study method is determined before its implementation, Whether the report is consistent with the plan; 3: Whether to explain the reasons for the study type; 4: Whether the search literature is comprehensive; 5: Whether the literature is independently screened by two people; 6: Whether the data is extracted by two people; 7: Whether to provide the list of documents and reasons for exclusion; 8: Whether to describe the basic characteristics of the included study in detail; 9: Whether the risk of included study bias is reasonably assessed; 10: Whether to report the source of research funding; 11: whether appropriate statistical methods are used to combine and analyze the results during the meta-analysis; 12: whether to consider the risk of bias in the meta-analysis or other evidence integration; 13: Whether the inclusion risk of study bias was considered when interpreting the results; 14: Whether the heterogeneous results are interpreted or discussed; 15: whether the publication bias was investigated during the quantitative synthesis, Whether to discuss its impact on the results; 16: Whether to report conflicts of interest and sources of funding.

According to the evaluation criteria, it can be rated “high,” “moderate,” “low” and “very low,” and 7 out of 16 items in the tool (2, 4, 7, 9, 11, 13 and 15) are critical areas.

#### 2.4.2 Risk of bias assessment

The Risk of Bias in Systematic Review (ROBIS), which aims at the bias risk of system evaluation, is not only used to evaluate the bias risk in the process of making and interpreting the results of multiple SRs/MAs such as intervention, diagnosis, etiology, and prognosis, etc., but also used to evaluate the correlation between the SRs/MAs problems and the practical problems to be solved by users. ROBIS scale was used in this overview to evaluate the risk of bias in the inclusion of SRs/MAs and the evaluation was carried out in three stages. ROBIS is useful for assessing the extent of bias in four domains: (1) Eligibility criteria for each study; (2) The identification and selection of studies; (3) Data collection and study appraisal; and (4) Overall synthesis and major findings. Within each domain, specific questions were used to determine the risk of bias, which was rated as “low,” “high,” or “unclear.”

#### 2.4.3 Report quality assessment

SR/MA is an important evidence to guide clinical practice. The clarity of its report affects the realization of its clinical value. Standard reports can reduce the bias between actual research results and published results, and increase the transparency of articles. The PRISMA Report Guide is designed to help authors improve the quality of their reports, obtain key information, and improve readability and credibility.

The quality of each SR/MA report for the included SRs/MAs was assessed by the PRISMA 2020 checklist, and each of the 27 items included in PRISMA 2020 was scored as “yes,” “partially yes” or “no.”

#### 2.4.4 Evidence quality assessment

In order to be useful to decision-makers, clinicians and patients, the SRs/MAs must provide not only the effect estimates of each result, but also the information needed to judge the correctness of these effect estimates. The Grading of Recommendations Assessment, Development, and Evaluation (GRADE) (19) provides a structure for SRs/MAs and clinical practice guidelines to ensure that it addresses all key issues of outcome evidence quality evaluation related to a specific issue in a consistent and systematic manner.

The quality of evidence for each SR/MA outcome was evaluated by the Grading of Recommendations Assessment, Development, and Evaluation (GRADE) system. Since the initial quality of evidence for RCTs is high, the quality of evidence for the outcomes of the study was evaluated based on downgrading factors such as limitations, inconsistencies, indirectness, imprecision, and publication bias of the study. According to the level of downgrade, they are rated as “high,” “moderate,” “low” and “very low.”

## 3 Results

### 3.1 Literature search and screening results

**Figure 1:**
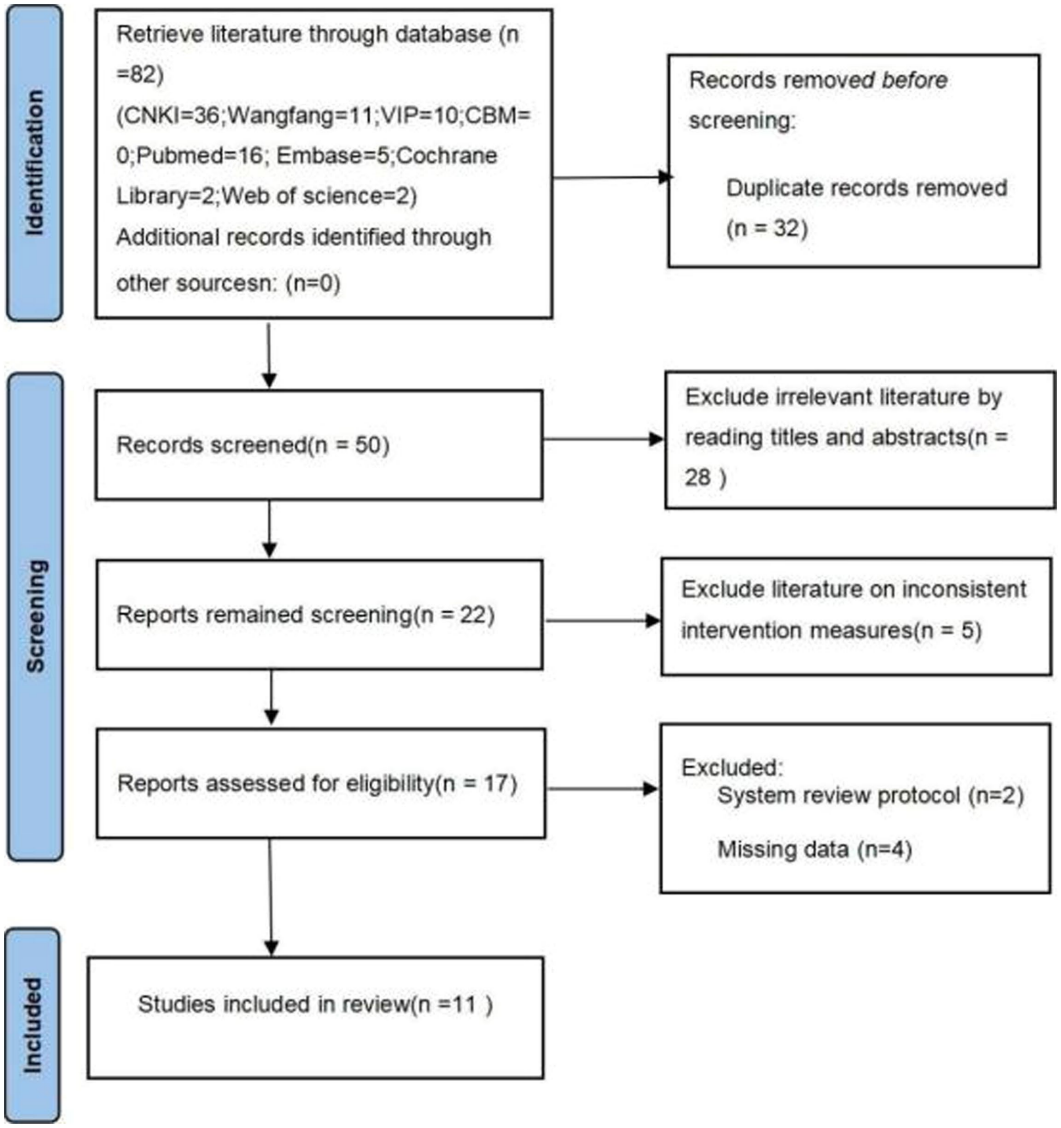
Flow diagram of literature screening process.

A total of 82 related literatures were retrieved, after 32 duplicate literatures were deleted, 28 unrelated literatures were deleted by reading titles and abstracts, then 5 literatures with inconsistent intervention measures were removed, and finally 6 literatures of systematic review protocols and literatures with missing data were deleted, 11 literatures (21–31) were finally included after layer-by-layer screening. The specific screening process is shown in Figure 1.

### 3.2 The basic characteristics of the included literature

Of the 11 included SRs/MAs, 2 of which were published in English and 9 in Chinese, all studies were conducted in China. The number of RCTs in the included SRs/MAs ranged from 5 to 27, with a sample size of between 750 and 1963. For quality evaluation, 6 (21, 22, 24, 28, 30, 31) SRs/MAs used Cochrane risk of bias tool, and 5 (23, 25–27, 29) SRs/MAs used the Jadad scale. The specific information is shown in Table 2.

**Table 2:** Basic information of the included SRs/MAs.

Author, year	Trials (subjects)	Intervention group	Control group	Quality assessment	Outcome indicators	Main results
Tu Y, 2022 (21)	27 (1293)	Acupuncture/ acupuncture + Routine treatment in western medicine /acupuncture + rehabilitation training	Routine treatment in western medicine /rehabilitation training	Cochrane	①, ②, ③, ④, ⑤, ⑦	Acupuncture provides an effective and safe treatment for patients with flaccid hemiplegia after stroke.
Li HP, 2022 (22)	25 (1963)	Acupuncture + basic treatment + rehabilitation training	Basic treatment + rehabilitation training	Cochrane	①, ②, ③, ④, ⑤, ⑥, ⑨	Acupuncture can effectively improve the upper limb hemiplegia spasm after stroke.
Zhu JM, 2021 (23)	21 (1524)	Acupuncture +rehabilitation training	Rehabilitation training	Jadad	①, ②, ④, ⑤	Clinical observation of acupuncture treatment after stroke.
Fan W, 2020 (24)	36 (2628)	Acupuncture +rehabilitation training	Rehabilitation training	Cochrane	①, ②, ④	Acupuncture may be a safe and effective adjuvant therapy.
Gou Y, 2019 (25)	8 (765)	Acupuncture +buyang huanwu decoction	Buyang huanwu decoction	Jadad	②, ⑤	The combination of acupuncture and buyang huanwu decoction can improve the daily activity ability of stroke patients with hemiplegia.
Wang YY, 2017 (26)	10 (1032)	Acupuncture + Routine treatment in western medicine / + rehabilitation training	Routine treatment in western medicine / rehabilitation training	Jadad	⑤	Acupuncture for stroke hemiplegia is more effective than other treatments.
Wang YQ, 2016 (27)	10 (783)	Acupuncture + Routine treatment in western medicine /acupuncture+ rehabilitation training	Routine treatment in western medicine/ rehabilitation training	Jadad	⑤	The therapeutic results of acupuncture for stroke hemiplegia have obvious advantages.
Jin D, 2016 (28)	12 (1228)	Acupuncture +rehabilitation training+Routine treatment in western medicine	Rehabilitation training +Routine treatment in western medicine	Cochrane	①, ②	Acupuncture treatment has superior advantages in improving the daily living ability of stroke patients with hemiplegia.
Song Y, 2016 (29)	5 (750)	Acupuncture +rehabilitation training+ Routine treatment in western medicine	Rehabilitation training +Routine treatment in western medicine	Jadad	①, ⑧, ⑨	Acupuncture combined with rehabilitation training can restore the lower limb motor function in stroke patients with hemiplegia.
Lin MQ, 2015 (30)	22 (1519)	Acupuncture	Routine treatment in western medicine / rehabilitation training	Cochrane	①	Acupuncture treatment of stroke has a certain effect on promoting their recovery.
Li N, 2002 (31)	9 (1253)	Acupuncture	Routine treatment in western medicine	Cochrane	③, ⑨	Acupuncture is effective in the treatment of stroke and hemiplegia.

① fugl-meyer as-sessment of low limb(FMA-L); ② Barthel Index(BI); ③ Clinic Neurological Function Deficit Scale (NDS); ④ The Modified Ashworth Spasticity Rating Scale score (MAS); ⑤ Clinical effectiveness; ⑥ The Clinical Spasticity Index score (CSI); ⑦ The Berg Balance Scale score; ⑧ functional comprehensive assessment score(FCA); ⑨ Brunnstorm stage score.

### 3.3 Results on SRs/MAs quality assessment

#### 3.3.1 Results of the methodological quality

The included SRs/MAs was assessed for its methodological quality by the AMSTAR-2. The results showed that the quality of the 9 included SRs/MAs were very low, since none of the SRs/MAs included in the quality assessment met the requirement of key Item 7 (none of the SRs/MAs provided an exclusion list). A summary of the results is shown in Table 3.

**Table 3:** Result of the AMSTAR-2 assessments.

Author, year	1	2*	3	4*	5	6	7*	8	9*	10	11*	12	13*	14	15*	16	Quality
Tu Y, 2022 (21)	Y	Y	Y	Y	Y	Y	N	Y	Y	Y	Y	Y	Y	N	Y	Y	L
Li HP, 2022 (22)	Y	N	Y	PY	Y	Y	N	Y	Y	Y	Y	Y	Y	Y	N	Y	VL
Zhu JM, 2021 (23)	Y	N	Y	PY	Y	Y	N	Y	Y	Y	Y	Y	Y	N	N	Y	VL
Fan W, 2020 (24)	Y	Y	Y	Y	Y	Y	N	Y	Y	Y	Y	Y	Y	N	Y	Y	L
Gou Y, 2019 (25)	Y	N	Y	PY	Y	Y	N	PY	Y	Y	Y	Y	Y	Y	N	Y	VL
Wang YY, 2017 (26)	Y	N	Y	PY	Y	N	N	PY	Y	N	Y	Y	Y	Y	N	N	VL
Wang YQ, 2016 (27)	Y	N	Y	PY	Y	Y	N	PY	Y	Y	Y	Y	Y	Y	N	Y	VL
Jin D, 2016 (28)	Y	N	Y	PY	Y	Y	N	Y	Y	Y	Y	Y	Y	Y	N	Y	VL
Song Y, 2016 (29)	Y	N	Y	PY	Y	Y	N	Y	Y	N	Y	N	N	N	N	N	VL
Lin MQ, 2015 (30)	Y	N	Y	PY	Y	Y	N	Y	Y	Y	Y	Y	Y	N	Y	Y	VL
Li N, 2002 (31)	Y	N	Y	PY	Y	Y	N	Y	Y	N	Y	N	N	N	N	N	VL

Y, yes; PY, partial yes; N, no; VL, very low; L, low; H, high. Q2, Q4, Q7, Q9, Q11, Q13, and Q15 are key areas.

#### 3.3.2 Results of the risk of bias assessment

For ROBIS, Phase 1 assesses the relevance of the research topic, and all SRs/MAs were rated as low risk of bias. In the Phase 2: Domain 1 assessed the study eligibility criteria, and all SRs/MAs were rated as low risk of bias; Domain 2 assessed the identification and selection studies, and 3 SRs/MAs were rated as low risk of bias; Domain 3 assessed the collection and study appraisal, 3 SRs/MAs were rated as high risk of bias; and Domain 4 assessed the synthesis and findings, and 8 SRs/MAs were rated as high risk of bias. Phase 3 considered the overall risk of bias in the reviews, and all SRs/MAs were rated as high risk of bias. The ROBIS scale evaluation results are shown in Table 4.

**Table 4:** Results of the ROBIS assessments.

Author, year	Phase 1	Phase 2				Phase 3
	Assessing relevance	Domain 1: Study eligibility criteria	Domain 2: Identification and selection of studies	Domain 3: Collection and study appraisal	Domain 4: Synthesis and findings	Risk of bias in the review
Tu Y, 2022 (21)	L	L	H	L	L	H
Li HP, 2022 (22)	L	L	L	H	H	H
Zhu JM, 2021 (23)	L	L	H	L	L	H
Fan W, 2020 (24)	L	L	H	L	L	H
Gou Y, 2019 (25)	L	L	L	L	H	H
Wang YY, 2017 (26)	L	L	H	L	H	H
Wang YQ, 2016 (27)	L	L	L	L	H	H
Jin D, 2016 (28)	L	L	H	H	H	H
Song Y, 2016 (29)	L	L	H	L	H	H
Lin MQ, 2015 (30)	L	L	H	L	H	H
Li N, 2002 (31)	L	L	H	H	H	H

L, low risk; H, high risk.

#### 3.3.3 Reporting quality of the included SRs/MAs

Reporting quality of the included SRs/MAs was evaluated as per the PRISMA checklist.6 of these items did not report at 100%. Mainly concentrated in the reporting rate of item 5 (protocol and registration) was 18%, the reporting rates of item 8 (search) and item 15 (study bias) were 82%, the item 27 (funding) reporting rate was 73%. The results of the PRISMA checklist assessment are shown in Table 5.

**Table 5:** Results of the PRISMA checklist.

Items	Tu Y, 2022 (21)	Li HP, 2022 (22)	Zhu JM, 2021 (23)	Fan W, 2020 (24)	Gou Y, 2019 (25)	Wang YY, 2017 (26)	Wang YQ, 2016 (27)	Jin D, 2016 (28)	Song Y, 2016 (29)	Lin MQ, 2015 (30)	Li N, 2002 (31)	Number of yes and partially yes (%)
Q1. Title	Y	Y	Y	Y	Y	Y	Y	Y	Y	Y	Y	100
Q2. Structured summary	Y	PY	PY	Y	PY	PY	PY	PY	PY	PY	PY	100
Q3. Rationale	Y	Y	Y	Y	Y	Y	Y	Y	Y	Y	Y	100
Q4. Objectives	Y	Y	Y	Y	Y	Y	Y	Y	Y	Y	Y	100
Q5. Protocol and registration	Y	N	N	Y	N	N	N	N	N	N	N	18
Q6. Eligibility criteria	Y	Y	Y	Y	Y	Y	Y	Y	Y	Y	Y	100
Q7. Information sources	Y	Y	Y	Y	Y	Y	Y	Y	Y	Y	Y	100
Q8. Search	Y	Y	Y	Y	Y	PY	Y	N	N	Y	Y	82
Q9. Study selection	Y	Y	Y	Y	Y	Y	Y	Y	Y	Y	Y	100
Q10. Data collection process	Y	Y	Y	Y	Y	Y	Y	Y	Y	Y	Y	100
Q11. Data items	Y	Y	Y	Y	Y	Y	Y	PY	PY	Y	PY	100
Q12. Risk of bias in individual studies	Y	Y	Y	Y	Y	Y	Y	Y	Y	Y	Y	100
Q13. Summary measures	Y	Y	Y	Y	Y	Y	Y	Y	Y	Y	Y	100
Q14. Synthesis of results	Y	Y	Y	Y	Y	Y	Y	Y	Y	Y	Y	100
Q15. Risk of bias across studies	PY	PY	PY	PY	PY	PY	PY	N	PY	PY	N	82
Q16. Additional analyses	Y	Y	Y	Y	Y	Y	Y	PY	Y	Y	N	91
Q17. Study selection	Y	Y	Y	Y	Y	PY	PY	PY	Y	Y	PY	100
Q18. Study characteristics	Y	Y	Y	Y	Y	Y	Y	Y	Y	Y	Y	100
Q19. Risk of bias within studies	Y	Y	Y	Y	Y	Y	Y	Y	Y	Y	Y	100
Q20. Results of individual studies	Y	Y	Y	Y	Y	Y	Y	Y	Y	Y	Y	100
Q21. Synthesis of results	Y	Y	Y	Y	Y	Y	Y	Y	Y	Y	Y	100
Q22. Risk of bias across studies	Y	Y	Y	Y	Y	Y	Y	Y	Y	Y	Y	100
Q23. Additional analysis	Y	Y	Y	Y	Y	Y	N	PY	PY	Y	N	82
Q24. Summary of evidence	Y	Y	Y	Y	Y	Y	PY	PY	Y	Y	PY	100
Q25. Limitations	Y	Y	Y	Y	Y	Y	Y	Y	Y	Y	Y	100
Q26. Conclusions	Y	Y	Y	Y	Y	Y	Y	Y	Y	Y	Y	100
Q27. Funding	Y	Y	Y	Y	Y	Y	N	N	Y	Y	N	73

Y, yes; N, no; PY, partial yes.

#### 3.3.4 Results of the quality of the evidence

A meta-analysis of the 42 outcomes in the study was performed, and the GRADE system was used to assess the quality of each outcome.1 outcome measure was high evidence quality, 14 moderate, 14 low and 13 very low evidence quality. Longitudinal analysis was performed for each degradation factor while horizontal evaluation was performed for the included systematic review/meta-analysis outcome indicators. We found that inconsistency (*n* = 29) was the main factor in reducing the quality of evidence, followed by publication bias (*n* = 28) and limitation (*n* = 23). Imprecision (*n* = 10) also has an impact on the evidence quality of the article. The results of evidence quality are shown in Table 6.

**Table 6:** Results of evidence quality.

Author, year	Outcomes			Studies (participants)	Limitations	Inconsistency	Indirectness	Imprecision	Publication bias	Relative effect (*95% CI)	Quality
Tu Y, 2022 (21)	FMA-L		Acupuncture vs. Rehabilitation	1(68)	−1^①^	−1^②^	0	−1^③^	−1^④⑤^	MD = 13.53 (11.65, 14.41)	VL
			Acupuncture + Rehabilitation vs. Rehabilitation	12(1091)	0	−1^②^	0	0	0	MD = 9.17, (5.84, 12.5)	M
			Acupuncture vs. Routine treatment in Western medicine	4(438)	0	0	0	0	−1^⑤^	MD = 16.86 (15.89, 17.84)	M
	BI		Acupuncture vs. Non-acupuncture therapy	1(40)	−1^①^	−1^②^	0	−1^③^	−1^④⑤^	MD = 10.00 (4.64, 15.36)	VL
			Acupuncture vs. Rehabilitation	1(68)	−1^①^	−1^②^	0	−1^③^	−1^④⑤^	MD = 32.21 (30.67, 33.75)	VL
			Acupuncture + Rehabilitation vs. Rehabilitation	12(1085)	0	−1^②^	0	0	0	MD = 11.35 (8.12, 14.57)	M
			Acupuncture vs. Routine treatment in Western medicine	3(322)	0	−1^②^	0	0	−1^⑤^	MD = 1.51 (1.16, 1.87)	L
	Clinical effectiveness		Acupuncture + Rehabilitation vs. Rehabilitation	4(317)	0	0	0	0	−1^⑤^	OR = 11.07 (5.78, 21.21)	M
			Acupuncture vs. Routine treatment in Western medicine	2(188)	−1^①^	−1^②^	0	−1^③^	−1^④⑤^	OR = 2.12 (1.19, 3.76)	VL
	NDs		Acupuncture + Rehabilitation vs. Rehabilitation	4(356)	0	−1^②^	0	0	−1^⑤^	MD = −3.52 (−6.32, −0.72)	L
			Acupuncture vs. Routine treatment in Western medicine	3(366)	0	−1^②^	0	0	−1^⑤^	MD = −1.88 (−2.31, −1.45)	L
	MAS		Acupuncture vs. Rehabilitation	1(68)	−1^①^	−1^②^	0	−1^③^	−1^④⑤^	MD = 3.71 (2.73, 4.69)	VL
	The Berg Balance Scale score			1(68)	−1^①^	−1^②^	0	−1^③^	−1^④⑤^	MD = 29.42 (23.85, 34.99)	VL
Li HP, 2022 (22)	FMA-L		Acupuncture + Non-acupuncture therapy vs. Non-acupuncture therapy	19(586)	0	−1^②^	0	0	−1^⑤^	SMD = 1.28 (0.89, 1.67)	L
	Clinical effectiveness			8(570)	0	−1^②^	0	0	0	OR = 3.30 (1.97, 5.52)	M
	MAS			8(569)	0	−1^②^	0	0	−1^⑤^	SMD = −0.50 (−0.90, −0.11)	L
	BI			15(1206)	0	0	0	0	−1^⑤^	SMD = 1.12 (0.74, 1.49)	M
	NDS			4(297)	0	0	0	0	−1^⑤^	WMD = −3.97 (−6.22, −1.72)	M
	Brunnstorm Stage score			2(120)	−1^①^	−1^②^	0	−1^③^	−1^④⑤^	SMD = 0.91 (0.53, 1.29)	VL
	CSI			2(142)	−1^①^	0	0	−1^③^	−1^④⑤^	SMD = −1.36 (−2.76, 0.03)	VL
Zhu JM, 2021 (23)	FMA-L		Acupuncture + Non-acupuncture therapy vs. Non-acupuncture therapy	20(1464)	0	−1^②^	0	0	−1^⑤^	MD = 7.59 (5.85, 9.33)	L
	Clinical effectiveness			18(1350)	0	0	0	0	0	OR = 3.65 (2.65, 5.02)	H
	MAS			8(575)	0	−1^②^	0	0	−1^⑤^	MD = −0.64 (−0.97, −0.30)	L
	BI			10(788)	0	−1^②^	0	0	−1^⑤^	MD = 9.96 (7.64, 12.27)	L
Fan W, 2020 (24)	FMA-L		Acupuncture + Non-acupuncture therapy vs. Non-acupuncture therapy	27(1875)	0	−1^②^	0	0	0	MD = 8.43 (6.57, 10.28)	M
	MAS			6(633)	0	−1^②^	0	0	0	MD = 0.46 (0.65, 0.27)	M
	BI			24(1782)	0	−1^②^	0	0	0	MD = 8.32 (5.30, 11.35)	M
Gou Y, 2019 (25)	Clinical effectiveness		Acupuncture + Non-acupuncture therapy vs. Non-acupuncture therapy	8(765)	−1^②^	0	0	0	0	OR = 3.89 (2.58, 5.86)	M
	BI			2(270)	−1^②^	−1^②^	0	0	−1^④⑤^	SMD = 2.35 (2.04, 2.66)	VL
Wang YY, 2017 (26)	Clinical effectiveness		Acupuncture + Non-acupuncture therapy vs. Non-acupuncture therapy	10(1032)	−1^②^	0	0	0	0	RR = 4.62 (1.12, 1.24)	M
Wang YQ, 2016 (27)	Clinical effectiveness		Acupuncture + Non-acupuncture therapy vs. Non-acupuncture therapy	10(783)	−1^②^	−1^②^	0	0	0	RR = 1.28 (1.19, 1.37)	L
Jin D, 2016 (28)	FMA-L		Acupuncture + Non-acupuncture therapy vs. Non-acupuncture therapy	8(678)	−1^②^	−1^②^	0	0	0	OR = 12.85 (10.69, 15.02)	L
	BI			8(748)	−1^②^	−1^②^	0	0	0	OR = 11.71 (9.51, 13.90)	L
Song Y, 2016 (29)	FMA-L		Acupuncture + Non-acupuncture therapy vs. Non-acupuncture therapy	5(750)	−1^②^	0	0	0	−1^⑤^	WMD = 5.96 (5.77, 6.15)	L
	FCA			5(750)	−1^②^	0	0	0	−1^⑤^	WMD = 2.6 (2.60, 2.72)	L
	Brunnstorm Stage score			5(750)	−1^②^	0	0	0	−1^⑤^	WMD = 1.06 (0.94, 1.18)	L
Lin MQ, 2015 (30)	FMA-L	Acupuncture vs. Non-acupuncture therapy	Acute stage (≤1 month)	6(410)	−1^②^	0	0	0	0	WMD = 6.71 (5.51, 7.90)	M
			Recovery time(≥1 month and ≤ 6 months)	3(101)	−1^②^	−1^②^	0	−1^③^	0	WMD = 2.50 (1.49, 3.50)	VL
			After the legacy period (≥6 months)	1(40)	−1^②^	−1^②^	0	−1^③^	−1^④⑤^	WMD = 2.89 (1.00, 4.78)	VL
			Spastic period (There is no limit to cycle)	3(393)	−1^②^	0	0	0	−1^⑤^	WMD = 1.60 (0.92, 2.28)	M
Li N, 2002 (31)	Brunnstorm Stage score		Acupuncture vs. routine treatment in Western medicine	5(618)	−1^②^	−1^②^	0	0	−1^④⑤^	OR = 2.45 (1.62, 3.72)	VL
	NDS			4(635)	−1^②^	−1^②^	0	0	−1^④⑤^	OR = 2.90 (1.98, 4.26)	VL

① The included studies have a large bias in methodology such as randomization, allocation concealment, and blinding. ② The confidence interval overlaps less or the I^2^ value of the combined results was larger. ③ The sample size from the included studies does not meet the optimal sample size or the 95% confidence interval crosses the invalid line. ④ The funnel chart is asymmetry. ⑤ Fewer studies were included, and their results were all positive, which may result in a large publication bias. *The 95% confidence interval does not cross. FMA-L, fugl-meyer as-sessment of low limb; BI, Barthel Index; NDS, Clinic Neurological Function Deficit Scale; MAS, The Modified Ashworth Spasticity Rating Scale score; CSI, The Clinical Spasticity Index score; FCA, functional comprehensive assessment score. H, High; M, Moderate; L, Low; VL, Very Low.

#### 3.3.5 Summary of results

The outcome measures extracted from the included studies are listed in Table 6.

##### 3.3.5.1 BI

6 SRs/MAs (21–25, 28) reported the BI. 1 SR/MA (21) conducted subgroup analyses, yielding the following results: (1) The therapeutic efficacy of acupuncture as a standalone treatment was superior to that of other conventional rehabilitation therapies; (2) The therapeutic efficacy of acupuncture as a standalone treatment was superior to non-acupuncture treatments; (3) The combined application of acupuncture and other conventional rehabilitation treatments demonstrated superior therapeutic efficacy compared to other conventional rehabilitation treatments; (4) Acupuncture as a standalone treatment exhibited superior therapeutic efficacy compared to conventional western medical treatments. Furthermore, 5 SRs/MAs (22–25, 28) reported that the combined application of acupuncture and non-acupuncture treatments showed superior therapeutic efficacy compared to non-acupuncture treatments.

##### 3.3.5.2 Clinical effectiveness

6 SRs/MAs (21–23, 25–27) reported the Clinical effectiveness. 1 SR/MA (21) conducted subgroup analyses, revealing the following results: (1) The combined application of acupuncture and other conventional rehabilitation treatments demonstrated superior therapeutic efficacy compared to other conventional rehabilitation treatments; (2) Acupuncture as a standalone treatment exhibited superior therapeutic efficacy compared to conventional western medical treatments. Furthermore, 5 SRs/MAs (22, 23, 25–27) reported that the combined application of acupuncture and non-acupuncture treatments showed superior therapeutic efficacy compared to non-acupuncture treatments.

##### 3.3.5.3 NDs

3 SRs/MAs (21, 22, 31) reported the NDs. 1 SR/MA (21) conducted subgroup analyses, yielding the following results: (1) The combined application of acupuncture and other conventional rehabilitation treatments demonstrated superior therapeutic efficacy compared to other conventional rehabilitation treatments; (2) Acupuncture as a standalone treatment exhibited superior therapeutic efficacy compared to conventional western medical treatments. Additionally, 1 SR/MA (22) reported that the combined application of acupuncture and non-acupuncture treatments showed superior therapeutic efficacy compared to non-acupuncture treatments. Furthermore, 1 SR/MA (31) reported that acupuncture as a standalone treatment exhibited superior therapeutic efficacy compared to conventional western medical treatments.

##### 3.3.5.4 MAS

4 SRs/MAs (21–24) reported the MAS. 1 SR/MA (21) reported that acupuncture as a standalone treatment demonstrated superior efficacy compared to other conventional rehabilitation therapies. 3 SRs/MAs (22–24) reported that the combined approach of acupuncture with non-acupuncture treatments exhibited superior efficacy over non-acupuncture treatments alone.

##### 3.3.5.5 The Berg Balance Scale score

1 SR/MA (21) reported the Berg Balance Scale score. The results indicated that the efficacy of acupuncture as a standalone treatment surpassed that of other conventional rehabilitation therapies.

##### 3.3.5.6 FMA-L

7 SRs/MAs (21–24, 28–30) reported the FMA-L. 1 SR/MA (21) conducted subgroup analyses, yielding the following results: (1) The therapeutic efficacy of acupuncture as a standalone treatment was superior to that of other conventional rehabilitation therapies; (2) The combined application of acupuncture and other conventional rehabilitation treatments demonstrated superior therapeutic efficacy compared to using other conventional rehabilitation treatments alone; (3) Acupuncture as a standalone treatment exhibited superior therapeutic efficacy compared to conventional western medical treatments. 5 SRs/MAs (22–24, 28, 29) reported that the combined approach of acupuncture with non-acupuncture treatments exhibited superior efficacy compared to non-acupuncture treatments alone. Additionally, 1 SR/MA (30) reported that acupuncture as a standalone treatment demonstrated superior efficacy compared to non-acupuncture treatments.

##### 3.3.5.7 Brunnstorm Stage score

3 SRs/MAs (22, 29, 31) reported the Brunnstorm Stage score. 2 SRs/MAs (22, 29) reported that the combined approach of acupuncture with non-acupuncture treatments exhibited superior efficacy compared to non-acupuncture treatments alone. Additionally, 1 SR/MA (31) reported that acupuncture as a standalone treatment demonstrated superior efficacy compared to conventional western medical treatments.

##### 3.3.5.8 CSI

1 SR/MA (22) reported the CSI. The results indicate that the combined approach of acupuncture with non-acupuncture treatments shows superior efficacy compared to non-acupuncture treatments alone.

##### 3.3.5.9 FCA

1 SR/MA (29) reported the FCA. The results indicate that the combined approach of acupuncture with non-acupuncture treatments shows superior efficacy compared to non-acupuncture treatments alone.

## 4 Discussion

High-quality SRs/MAs is an important source for evidence-based medicine to obtain the best evidence, and is recognized as the cornerstone for evaluating clinical efficacy and formulating clinical guidelines and specifications (20). Overview of SRs/MAs is a comprehensive research method to comprehensively collect the treatment and diagnosis of the same disease or health problems, which can provide more concentrated users with high-quality evidence and provide better guidance for future clinical work (32, 33). In this study, the included 11 SRs/MAs of acupuncture for stroke hemiplegia were reevaluated, and the included articles were evaluated by multiple quality evaluation methods to provide high-quality evidence-based evidence and decision basis for the clinical efficacy of acupuncture for hemiplegia, and provide further reference and evidence support for future clinical applications. At present, acupuncture has been widely used for the treatment of hemiparesis caused by stroke. When stroke patients are in a hemiparetic state, acupuncture can promote the recovery of muscle strength and muscle tone, improve limb motor function, and effectively prevent various complications (34, 35). However, the current research on acupuncture therapy for stroke is extensive, with studies primarily focusing on spasticity and the recovery phase in investigating the mechanisms underlying acupuncture’s improvement of limb motor function.

In this study, the methodology of quality was evaluated by the included articles using AMSTAR-2, and all the 7 key items had some defects: (1) Only 2 of the 11 included research protocols were formulated in advance, which may affect the rigor of the systematic review formulation; (2) 9 articles do not manually search the grey literature or provide a complete search form, and cannot check whether the included literature search is comprehensive and accurate, and whether the data extraction is accurate and not reproducible; (3) None of the articles provides a list of excluded articles, which may reduce the credibility of the SRs/MAs; (4) 3 articles did not report any potential conflicts of interest or funding, which would bias the systematic evaluation. The absence of the above key items is the main factor leading to the lower results of the methodological quality evaluation.

The SRs/MAs bias risk results included in the ROBIS scale evaluation showed that incomplete literature search and data synthesis and incomplete presentation of results were the main factors leading to high bias. This study evaluated the quality of the report using the PRISMA checklist, which showed that lack of protocol registration, incomplete search strategy, and uncertainty about funding sources affected the rigor of the systematic overview as the highest evidence for diagnosis and treatment.

Quality grade evaluation of the included articles using the GRADE rating scale found that all the outcome measures were mostly of low quality (1) Some studies also have great risks in terms of heterogeneity and inaccuracy, mainly because the overlap of confidence intervals is too small or the sample size does not meet the optimal information sample size, or the confidence interval is too wide, which may be unreasonably related to the literature inclusion criteria and retrieval method; (2) More than half of the outcome indicators were downgraded, mainly because of the heterogeneity of the included SRs/MAs, and only a few conducted sensitivity and subgroup analyses; (3) In the evaluation of limitations, 23 outcome indicators were downgraded, because the randomization method and allocation of the included studies were not clear, only a small part of them described whether they was blind, and there was a high possibility of implementation and measurement bias; (4) Publication bias is also widespread.

To the best of our knowledge, this overview is the first article to use the SRs/MAs for clinical evaluation of acupuncture for stroke hemiplegia, which can provide comprehensive evidence reference for clinical practice. The assessment processes using tools such as AMSTAR-2, ROBIS, PRISMA, and grading have highlighted the limitations of SRs/MAs and RCTs, which can guide future high-quality clinical research. However, we must also acknowledge the limitations of this overview. Due to language restrictions, this study only included systematic reviews published in Chinese and English, and did not include Korean and Japanese databases that have similar backgrounds in traditional Chinese medicine research. Additionally, the search process actually overlooked manual searching, leading to some degree of selection bias. Furthermore, the literature screening and quality assessment were conducted by two researchers and were somewhat subjective. The number of included SRs/MAs was small, and the overall quality was not high.

### 4.1 Implications for future research

To reduce various biases such as selection bias, implementation bias, and measurement bias, further original studies should be conducted using large-sample, multicenter, long-term clinical randomized controlled trials based on evidence-based medicine standards. Attention should be given to properly and rationally implementing randomization, concealing allocation, and blinding. Additionally, to improve the quality of evidence, authors should register their study protocols before conducting SRs/MAs to ensure the rigor of their procedures. During the literature search and screening process, the excluded literature information and complete search strategies for all databases should be provided to ensure replicability. When quantitatively calculating effect sizes, individual study results should be systematically excluded one by one to ensure the stability of the results. Furthermore, a comprehensive assessment of publication bias will also improve the accuracy of the meta-analysis results. In order to develop a more effective treatment prescription and evaluation system, it is advisable for future studies to report detailed information regarding acupuncture treatment. This includes the number of needles used in each treatment session, specific acupuncture techniques employed, needle depth, needle reactions, treatment process, qualifications of the acupuncturist, years of experience of the assessors and clinicians involved, and the provision of a standardized and explicit treatment plan. Reporting these details will contribute to a better understanding of the acupuncture intervention and facilitate the replication and comparison of studies, ultimately leading to improved treatment outcomes and enhanced evaluation of acupuncture efficacy.

## 5 Conclusion

In conclusion, acupuncture treatment of stroke hemiplegia has certain efficacy, which can effectively improve the clinical manifestations of patients and reduce the disability rate. However, there are widespread problems of low methodological quality and low quality of evidence for SRs/MAs, which limits the reliability of the results and requires more high-quality original studies to provide evidence to support them.

## Data availability statement

The original contributions presented in the study are included in the article/supplementary materials, further inquiries can be directed to the corresponding author.

## Author contributions

MF: Writing – original draft, Writing – review & editing. BZ: Writing – original draft, Writing – review & editing, Methodology. CC: Data curation, Methodology, Writing – original draft. RL: Methodology, Validation, Writing – original draft. WG: Writing – original draft, Writing – review & editing.
